# Analysis of the Structure and the Thermal Conductivity of Semi-Crystalline Polyetheretherketone/Boron Nitride Sheet Composites Using All-Atom Molecular Dynamics Simulation

**DOI:** 10.3390/polym15020450

**Published:** 2023-01-14

**Authors:** Yuna Oh, Kwak Jin Bae, Yonjig Kim, Jaesang Yu

**Affiliations:** 1 Composite Materials Application Research Center, Institute of Advanced Composite Materials, Korea Institute of Science and Technology (KIST), Chudong-ro 92, Bongdong-eup, Wanju-gun 55324, Republic of Korea; 2 Department of Mechanical Design Engineering, Jeonbuk National University, Baekje-daero 567, Deokjin-gu, Jeonju 54896, Republic of Korea

**Keywords:** semi-crystalline polyetheretherketone, boron nitride sheet, thermal conductivity, polymer composite

## Abstract

Thermal transport simulations were performed to investigate the important factors affecting the thermal conductivity based on the structure of semi-crystalline polyetheretherketone (PEEK), and the addition of boron nitride (BN) sheets. The molecular-level structural analysis facilitated the prediction of the thermal conductivity of the optimal structure of PEEK reflecting the best parameter value of the length of amorphous chains, and the ratio of linkage conformations, such as loops, tails, and bridges. It was found that the long heat transfer paths of polymer chains were induced by the addition of BN sheets, which led to the improvement of the thermal conductivities of the PEEK/BN composites. In addition, the convergence of the thermal conductivities of the PEEK/BN composites in relation to BN sheet size was verified by the disconnection of the heat transfer path due to aggregation of the BN sheets.

## 1. Introduction

Polymers are widely used in many industrial fields, including the aerospace, automobile, semiconductor, and biomedical fields because they are light weight, easy to process, have low production costs, good mechanical properties, and chemical resistance [1,2,3,4]. Depending on their degree of crystallinity, polymers are classified as an amorphous polymer, a semi-crystalline polymer, or a crystalline polymer. Analysis of linkage conformations such as loops, tails, and bridges affecting the material properties of semi-crystalline polymers is important because the structure, mechanical properties, and thermal properties of the crystalline structure and the amorphous structure are different [5,6,7]. All-atom molecular dynamics (MD) simulation, which deals with all atoms of molecules of a simulation model, can facilitate a detailed analysis of material properties, such as the behavior of polymer chains, the radius of gyration, and the interaction between materials at the atomic level [8,9,10]. Zhu et al. [11] investigated the tensile property of semi-crystalline polyurethane using an MD simulation. The tensile yield stress of the model with many bridge chains connecting the crystalline and amorphous domains was 19 times higher than that of a model with no bridge due to the straightening and stretching of the bridge chain. In addition, He et al. [12] reported that the thermal conductivity of the interphase region in the semi-crystalline polyethylene was determined by the stretching of the bridge chains. While the effects of bridge chains on material properties have been analyzed in many studies, the effect of the other linkage conformations of loops and tails on the material properties have not yet been concretely considered in MD simulations. We found that the thermal conductivity of a semi-crystalline polymer depends on the contents of the linkage conformations of the loops, tails, and bridges. Therefore, the effect of linkage conformation on thermal properties was investigated to determine the ideal structure of a semi-crystalline polymer with high thermal conductivity. Among semi-crystalline polymers, semi-crystalline polyetheretherketone (PEEK) is a high-performance engineering thermoplastic with excellent wear resistance and heat resistance. PEEK with a low thermal conductivity of 0.25 W/mK was used to investigate the ratio of the optimal combination of loops, tails, and bridges, which leads to the improvement of the thermal conductivity [13].

Adding fillers is a general approach for improving the thermal properties of polymers among many methods, such as the crystallite orientation of polymer and increasing crystallinity [14,15,16]. A boron nitride (BN) sheet has been used as a filler in polymer composites for various applications, including aerospace, electronic devices, and semiconductors, because it leads to excellent mechanical properties and high thermal transport performance [17,18]. In a report by Ghosh et al. [19], the thermal conductivity of PEEK increased after the addition of hexagonal boron nitride (h-BN). The thermal conductivities of polymer composites reinforced with BN sheets were higher than those of untreated polymers due to the effect of not only the high thermal conductivities of the fillers, but also the increase of phonon velocities between the polymer chains and fillers [20]. Accordingly, the factor responsible for increasing the phonon mobility should be investigated to improve the thermal conductivity of the composite. In addition, it is known that the thermal conductivity of a polymer composite depends on the arrangement of the polymer chains. Li et al. [21] reported that the thermal conductivity of the poly(dimethylsiloxane)/h-BN composite was improved because the arrangement of polymer chains completely covered the surface of the h-BN sheet, inducing strong phonon mobility. The factors affecting the distribution of polymer chains should be investigated because the arrangement of polymer chains on the surface of a BN sheet is related to the thermal conductivity of the composite. Although the thermal conductivity of the composite increased with the addition of fillers, it converged with the aggregation of the fillers [22,23]. The factor inducing the aggregation of fillers should be investigated to understand the reason for the convergence of the thermal conductivities of the composites.

In this study, a thermal transport simulation was performed to investigate thermal conductivity in relation to the ratio of the linkage conformations between the crystalline domain and amorphous domain linked by loops, tails, and bridges. The optimal chain length of the amorphous PEEK was selected with the length at the point of convergence of the thermal conductivities of the semi-crystalline PEEK models. The interphase thermal conductivity between the crystalline domain and the amorphous domain, and the heat transfer path of the semi-crystalline PEEK were investigated after the thermal transport simulation, in accordance with the various combinations of loops, tails, and bridges. The thermal transport simulations of the semi-crystalline PEEK/BN composites reinforced with BN sheets were performed to verify the effect of the addition of BN sheets and the mobility of phonon between the BN sheets and PEEK chains. In addition, the factor inducing the aggregation of fillers in the PEEK/BN composite was investigated by visualizing the equilibrated simulation models.

## 2. Computational Methods

### 2.1. Semi-Crystalline Polyetheretherketone Modeling

**Figure 1:**
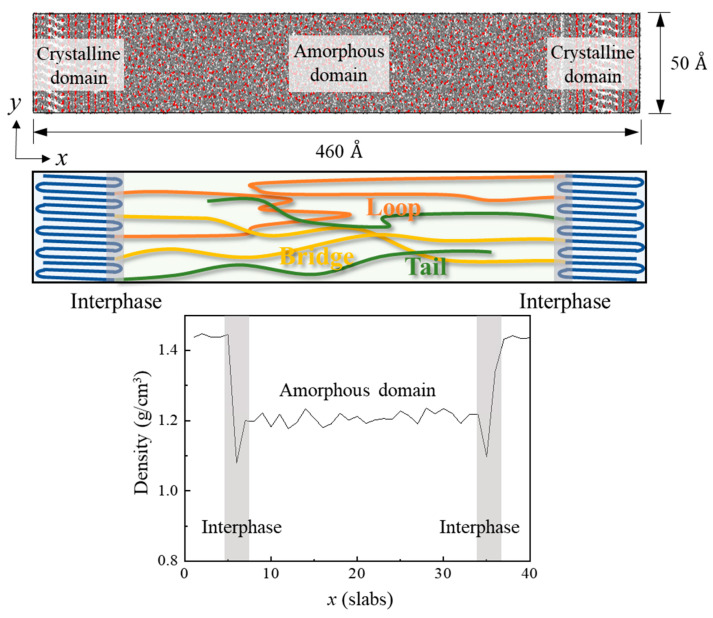
Structure and density of a semi-crystalline PEEK in the periodic boundary condition.

The semi-crystalline PEEK model consisted of a crystalline domain with aligned chains and an amorphous domain with randomly entangled chains in the periodic boundary system. The simulation model was constructed based on experimental results, as shown in Figure 1. The degree of crystallinity of the semi-crystalline PEEK was about 30%. It is identical to the experimental result obtained with a differential scanning calorimeter (DSC) [24]. The size of a crystalline PEEK domain is 124 Å (*x*) × 50 Å (*y*) × 50 Å (*z*). The atomic content of crystalline PEEK with 30,800 atoms, relative to the total number of atoms in the semi-crystalline PEEK, is 30%. The amorphous PEEK was set to 70% relative to the total number of atoms in the semi-crystalline PEEK. The total size of the semi-crystalline PEEK model was 460 Å (*x*) × 50 Å (*y*) × 50 Å (*z*) in the periodic boundary condition. The density distribution of the amorphous domain well matched the density of the amorphous PEEK, which was of 1.26 g/cm^3^ as obtained from the experiment [25]. The crystalline PEEK had a density of about 1.40 g/cm^3^ [25]. This was well matched with the density of the crystalline domain in this work. A range of 10 to 100 monomer units was used to verify the change in thermal conductivity for various chain lengths of amorphous PEEK. An analysis of thermal transport according to the ratio of the linkage conformation between the crystalline domain and the amorphous domain was performed using the selected chain length of amorphous PEEK. The crystalline domain and the amorphous domain of semi-crystalline PEEK were linked by loops, tails, and bridges (Figure 1). Both ends of a loop chain are connected to the same side of the crystalline domain. A tail chain only connects to one end of the crystalline domain. The bridge chain is connected to the crystalline domains by one polymer chain. The simulation models were constructed using Materials Studio 2017 software with a Dreiding force field [26].

The effects of the chain length of the amorphous PEEK and the contents of linkage conformations on thermal conductivity were analyzed following the thermal transport simulations of the semi-crystalline PEEK models. The radius of gyration (*R_g_*), which is based on the size of the molecule from the center of mass in a polymer chain, was calculated by

(1) $$R_{g}^{2}=\frac{1}{M}\sum^{\text{​}}m_{i}{\left(r_{i}-r_{cm}\right)}^{2}$$

where *M* is the total mass of the polymer chain, *m_i_* is the mass of the atom, *r_i_* is the coordinate of the atom, and *r_cm_* is the coordinate of the center of mass on a polymer chain. The radius of gyration indicates the distribution size of a single polymer chain. The *R*_a,g_ is the average value of the radius of gyration of the amorphous polymer chains. In the simulation model, a large *R*_a,g_ means a long heat transfer path. The radius of gyration on the bulk polymer (*R_bulk_*) in the amorphous domain was calculated by

(2) $$R_{bulk}^{2}=\frac{1}{M_{b}}\sum^{\text{​}}m_{i}{\left(r_{i}-r_{cm,bulk}\right)}^{2}$$

where *M_b_* is the total mass of the amorphous polymer chains and *r*_*cm*,*bulk*_ is the coordinate of the center of mass on the bulk amorphous polymer. In the simulation model, *R_bulk_* is the size of the bulk polymer from the center of mass on the bulk polymer to all amorphous polymer chains. *R_bulk_* indicates the degree of entanglement of the polymer chains. A large *R_bulk_* means that amorphous polymer chains are widely distributed with low entanglement. BN sheets were used to improve the thermal property of the semi-crystalline PEEK. In our previous study [27], the newly proposed Dreiding force field (N-DFF) was developed to calculate the potential energy of boron and nitride atoms using all-atom MD simulation. The force constants of the N-DFF were parameterized based on the potential energy obtained from the density functional theory (DFT). Consequently, the N-DFF can be used to help accurately predict the material properties of the BN sheet, such as its mechanical and thermal properties. In this work, the N-DFF was used to analyze the thermal transport of the PEEK/BN composite. The thermal properties of the semi-crystalline PEEK were calculated using the original Dreiding force field (DFF) because the DFF provides a more accurate prediction of material properties when the polymers consist of carbon, oxygen, and hydrogen atoms [8,28]. In addition, the parameters of the bond stretching terms of the B–H and N–H bonds were obtained using the linear least square fitting (LLSF) method for the accurate force constants of the bonded term. A detailed description of the calculation method can be found in the Supplementary Materials.

### 2.2. Thermal Transport Using Molecular Dynamics Simulation

**Figure 2:**
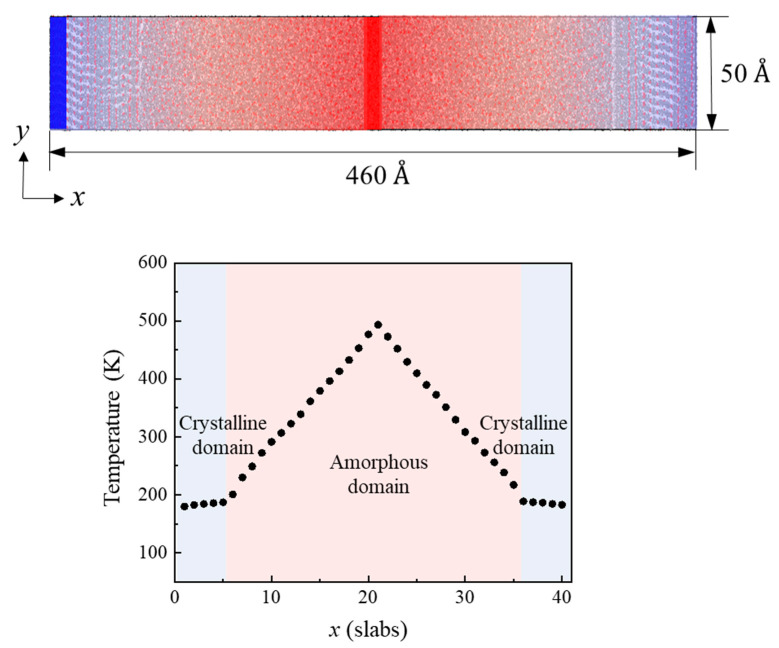
Temperature gradient of a semi-crystalline PEEK model during RNEMD simulation.

All of the simulation models were optimized using the conjugate gradient method to minimize the total potential energy. The optimized models were equilibrated using an isothermal–isobaric ensemble (NPT) at room temperature for 3 ns with a time step of 1 fs. After the equilibration process, the thermal conductivity was calculated using a reverse non-equilibrium molecular dynamics (RNEMD) simulation. A swap of kinetic energy between the atoms in a heat sink and the atoms in a heat source was performed under the microcanonical ensemble (NVE) for 3 ns (Figure 2). A temperature gradient was generated during the heat transfer process with a swap time of 100 fs. The semi-crystalline PEEK models and the composite models composed of the semi-crystalline PEEK and BN sheets were divided into 40 slabs along the *x* direction. The thermal conductivity (*K*) was calculated by

(3) $$J=\frac{1}{2tA}\underset{N_{swap}}{\sum }\frac{m_{h}v_{h}^{2}-m_{c}v_{c}^{2}}{2}$$

(4) $$K=\frac{J}{\partial T/\partial x}$$

where *J* is the total heat flux during the heat transfer time *t*, *A* is the cross-sectional area of the simulation model, *m_h_* and *m_c_* are the atomic masses of the heat source and the heat sink, respectively, and *v* is the velocity of the atom. The phonon behavior is closely related to the thermal transport in the materials. The phonon density of states (*PDOS*) of the simulation models was calculated using a Fourier transform of the velocity autocorrelation function (*VACF*). The *VACF* was averaged over the value of the BN atoms and PEEK atoms in the composite model obtained during the equilibration process. The *PDOS* was calculated by

(5) $$PDOS\left(\omega \right)=\frac{1}{\sqrt{2\pi }}\int_{0}^{\tau }VACF\left(t\right)e^{-i\omega t}dt$$

(6) VACF(t)=〈ν(t)ν(0)〉

where *ω* is the frequency, *τ* is a total integration time, and *ν*(*t*) is the velocity of atoms at time *t*. The angle bracket denotes the ensemble average of all atoms in the simulation model. All equilibration and thermal transport simulations were performed using the Large-scale Atomic/Molecular Massively Parallel Simulator (LAMMPS) [29].

## 3. Results and Discussion

### 3.1. Effect of the Chain Length of Amorphous PEEK on the Thermal Conductivity

**Figure 3:**
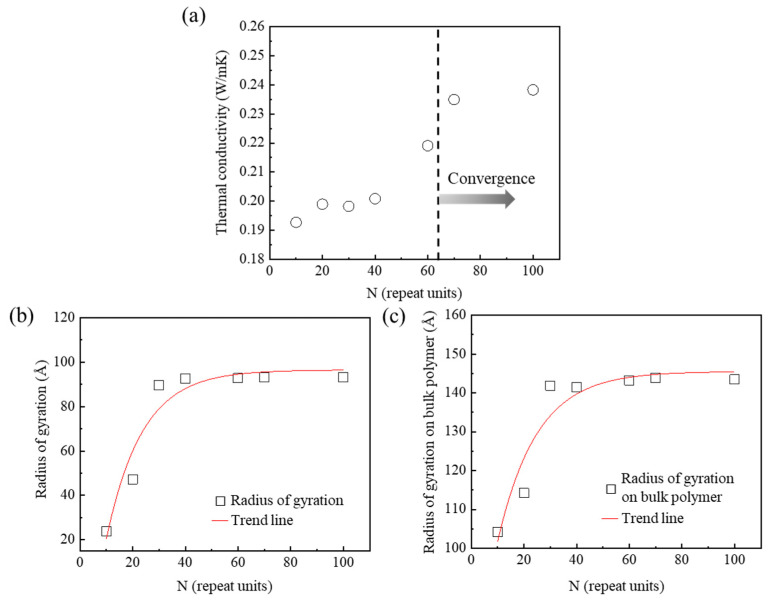
Thermal transport according to the length of the amorphous chains in semi-crystalline PEEK: (**a**) thermal conductivities, (**b**) radius of gyration, and (**c**) radius of gyration on the bulk polymer.

Thermal transport simulations of the semi-crystalline PEEK models for various chain lengths were performed to determine the optimal repeat unit of the amorphous chain. For example, PEEK10 was a semi-crystalline PEEK model consisting of a crystalline domain and the amorphous domain with an amorphous chain of 10 repeat units. Thermal conductivity was observed to increase from PEEK10 to PEEK70. It converged at about 0.24 W/mK when there were 70 chain repeat units (Figure 3a). The convergence of bending, torsion, and non-bonding potential energies on the polymer molecule according to the chain lengths led to the convergence of the thermal conductivities [10]. The thermal conductivity from the simulation matched the experimental value well. It was about 0.25 W/mK when the chain repeat units exceeded 70 [13]. The semi-crystalline PEEK model facilitated the reliable calculation of thermal conductivity. The thermal conductivity of PEEK100 with an *R*_a,g_ of about 93 Å was higher than that of PEEK10 because PEEK100 had a longer heat transfer path than PEEK10 (Figure 3b). The thermal conductivity of a polymer is associated with phonon transfer in a polymer chain. The thermal conductivity of PEEK100 with its long polymer chain was higher than that of PEEK10 with the short polymer chain, due to an increase of phonon mobility caused by the increase of bond vibration [30]. In addition, thermal transport is generated by collisions among the chains. The thermal conductivity of PEEK10 was lower than those of other models because of its low phonon mobility, induced by the reduction in collision between shorter chains compared with the chains of other models [31]. In addition, the entanglement of polymer chains affected the thermal conductivities of the PEEK models in this study. PEEK100 with an *R_bulk_* of about 143 Å had the highest thermal conductivity due to the increase of the heat transfer path induced by a reduction of entanglement of the polymer chains (Figure 3c). These results indicate that the heat transfer path on a polymer chain and the entanglement of polymer chains are both important factors affecting thermal conductivity. The chain with the 70 repeat units at the point of convergence was selected as an amorphous chain to investigate the effect of linkage conformation connecting the crystalline domain and the amorphous domain.

### 3.2. Effect of Loop and Tail Conformations Connecting the Crystalline Domain and the Amorphous Domain on Thermal Conductivity

**Figure 4:**
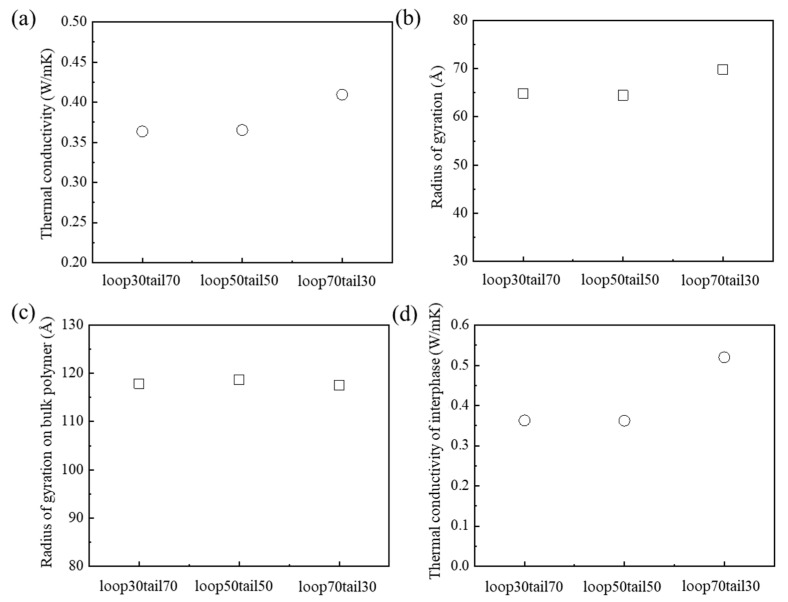
Thermal transport according to the ratio of loop and tail conformations: (**a**) thermal conductivities, (**b**) radius of gyration, (**c**) radius of gyration on the bulk polymer, and (**d**) thermal conductivities of interphase.

The thermal conductivities of PEEK models were predicted according to the various ratios of loop and tail conformations. The PEEK models were labeled loop30tail70, loop50tail50, and loop70tail30 models, according to the ratio of the linkage conformation on the amorphous chain. For example, loop30tail70 model was set to 30% loop and 70% tail conformations, relative to the total number of amorphous chains. The thermal conductivity of the loop70tail30 model, which had a high content of loop conformation, was about 0.41 W/mK (Figure 4a). This was about 14% higher than those of loop30tail70 and loop50tail50. The loop70tail30 model had the longest heat transfer path, induced by the largest *R*_a,g_ (Figure 4b). The *R_bulk_* values of three models were the same, about 118 Å (Figure 4c). This result means that the radial distribution of the bulk polymer did not change the thermal conductivities of the PEEK models in accordance with the ratio of loop and tail conformations. The thermal conductivity in the model, according to the ratio of loop and tail conformations, was related to the interphase thermal conductivity in the interphase region between the crystalline domain and the amorphous domain. The thermal conductivities of the loop30tail70, loop50tail50, and loop70tail30 models in the interphase region were about 0.36, 0.36, and 0.52 W/mK, respectively (Figure 4d). The thermal conductivity of the interphase region of loop30tail70 was lower than that of loop70tail30 due to the low heat transfer path induced by the tail chains, which are connected to the crystalline domain by only one end of the chain. On the other hand, the thermal conductivity of the interphase of the loop70tail30 model was about 44% higher than those of the other models due to the increase of the heat transfer path, because both ends of the loop chain were connected with the crystalline domain. Consequently, the thermal conductivity of the loop70tail30 model, with the high content of loops, was improved by the increase of the thermal conductivity of the interphase region. Therefore, the ratios of loop and tail conformations are important to improving the thermal conductivity of semi-crystalline PEEK.

### 3.3. Effect of Bridge Conformation, Connecting Two Sides of the Crystalline Domain on Thermal Conductivity

**Figure 5:**
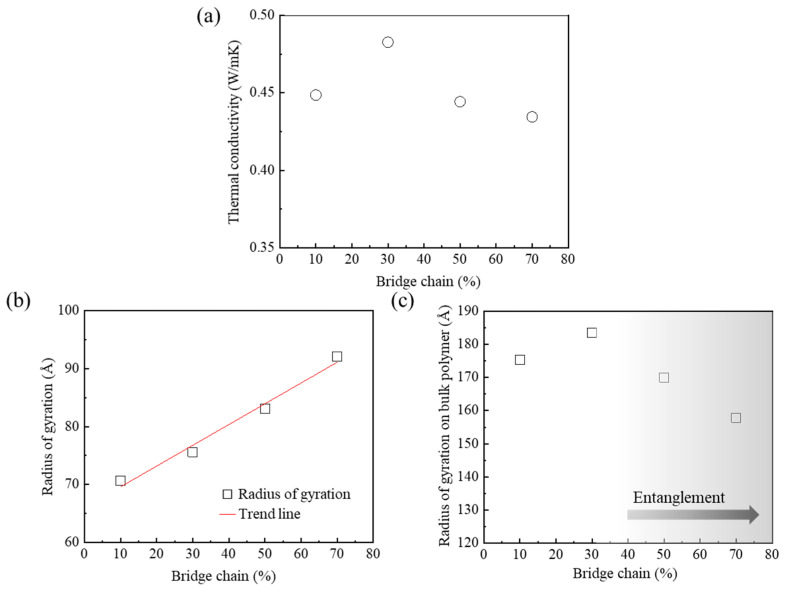
Thermal transport according to the content of bridge conformation: (**a**) thermal conductivities, (**b**) radius of gyration, and (**c**) radius of gyration on bulk polymer.

The thermal conductivities for various ratios of bridge conformations were investigated to determine the optimal content of bridge conformation for improving the thermal conductivity. The bridge conformation content was set to 10, 30, 50, and 70%, relative to the total number of amorphous chains. The residual content of amorphous chains, except for the bridge conformation, consists of loop and tail conformations. The ratio of loop and tail conformations in loop70tail30 was used to determine the optimal content of the bridge conformation, which needed to achieve the highest thermal conductivity of semi-crystalline PEEK. The thermal conductivity of the model with a bridge content of 30% was about 0.48 W/mK. This was higher than those of the other models (Figure 5a). The thermal conductivities of models with bridge contents in the range of 10% to 30% increased as the *R*_a,g_ values increased. Among the loop, tail, and bridge conformations, the bridge chain is the longest chain along the x-axis. Therefore, the *R*_a,g_ of the amorphous chains increased in a linear form as the bridge content increased (Figure 5b). In addition, the PEEK model with a bridge conformation of 30% had the highest thermal conductivity due to the long thermal transport path induced by the wide distribution of polymer chains with low entanglement (Figure 5c). However, thermal conductivity decreased when the bridge chain content was over 30%. This result is related to the entanglement of the polymer chains. The *R_bulk_* values of models with bridge chain contents more than 30% gradually decreased due to the increase of the entanglement of polymer chains. The thermal conductivity decreased due to excess phonon scattering, induced by the increased entanglement of polymer chains [32]. These results indicate that a bridge conformation of 30% of the PEEK model with low phonon scattering and low entanglement of polymer chains was the optimal content for improving thermal conductivity.

### 3.4. Effect of the Addition of BN Sheets on the Thermal Conductivity of the PEEK/BN Composite

**Figure 6:**
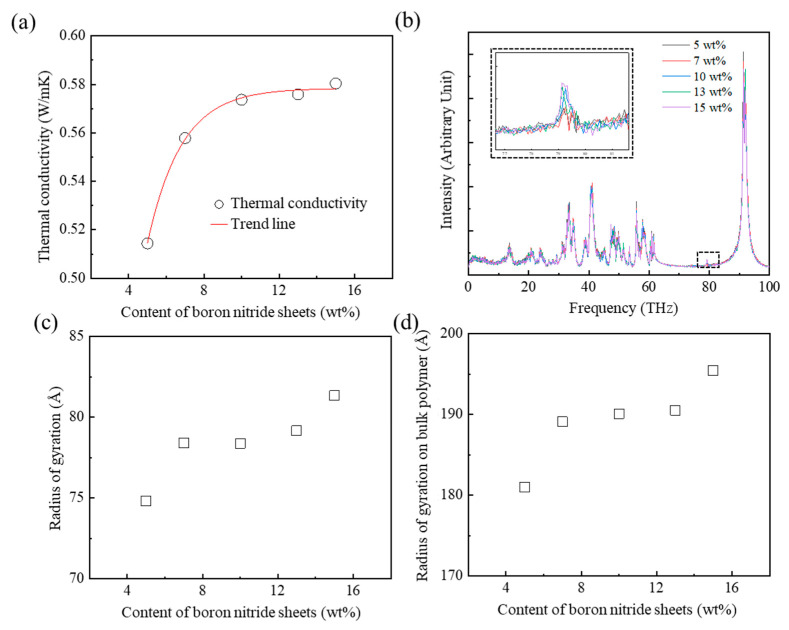
Thermal transport according to the content of boron nitride sheets in PEEK/BN composites: (**a**) thermal conductivities, (**b**) PDOS of composites, (**c**) radius of gyration, and (**d**) radius of gyration on the bulk polymer.

The thermal conductivities of the PEEK/BN composites with the addition of BN sheets were investigated to verify how the filler affected high thermal conductivity. The semi-crystalline PEEK model with a bridge conformation of 30% was used to construct the PEEK/BN composite model using square BN sheets with a lateral length of 45 Å. The thermal conductivities of the PEEK/BN composites gradually increased when the contents of the BN sheets increased from 5 wt% to 10 wt%. It converged at about 0.58 W/mK when the BN sheet contents exceeded 10 wt% (Figure 6a). The thermal conductivities of composites with BN sheets contents of more than 10 wt% were improved by about 14% compared to the composite with 5 wt% BN sheets. The increasing content of the BN sheets with high thermal conductivity improved the thermal conductivity of the PEEK/BN composite. Figure 6b shows the phonon behaviors of the PEEK/BN composites according to the BN sheet content. When the BN sheet content was over 7 wt%, the phonon intensities increased in the frequency region of 79 THz. This result indicates that heat transfer was actively promoted by the increase of phonon vibration within the BN sheets. As a result, the thermal conductivity increased as the phonon intensities in the BN sheets increased. Additionally, the thermal conductivity of the composite increased as phonon scattering was reduced by the many heat transfer pathways created between the polymer chains and fillers [33]. Figure 6c shows that the *R*_a,g_ increased with the addition of BN sheets. The *π*–*π* interaction between the aromatic rings of the polymer chains and the BN sheets facilitated the distribution of polymer chains on the surface of the BN sheets. As a result, the polymer chains became widely distributed along the surface of the BN sheets. Therefore, the long heat transfer path induced by the wide distribution of the polymer chains leads to an increase of thermal conductivity. In addition, the *R_bulk_* increased with the increasing BN sheet content (Figure 6d). This result indicates that the wide distribution of polymer chains induced by the increase of BN sheet content reduced the entanglement of the polymer chains. The reduction of entanglement led to a reduction of the phonon scattering in the PEEK/BN composite. These results indicate that the increase of phonon vibration, the reduction of phonon scattering, and the wide distribution of polymer chains with the addition of BN sheets led to the improvement of thermal conductivity on the PEEK/BN composite.

### 3.5. Effect of the Lateral Size of the Square BN Sheets on the Thermal Conductivity of the PEEK/BN Composite

**Figure 7:**
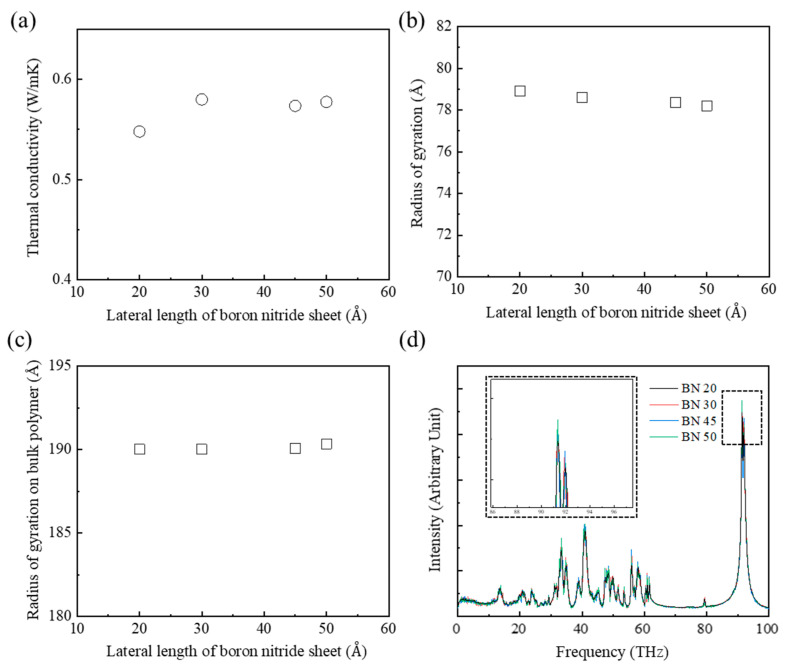
Thermal transport according to the lateral length of square BN sheets in the PEEK/BN composites: (**a**) thermal conductivities, (**b**) radius of gyration, (**c**) radius of gyration on the bulk polymer, and (**d**) PDOS of composites.

Thermal transport simulations of the composites were performed to investigate the thermal conductivities for various lateral sizes of square BN sheets. The total BN sheet content was selected to be the 10 wt%. This was the point of convergence of the thermal conductivities. The lateral lengths of a square BN sheet ranged from 20 Å to 50 Å. The thermal conductivities of PEEK/BN composites with BN sheets with lateral lengths of more than 30 Å were improved by about 6% compared to the model with BN sheets with lateral lengths of 20 Å (Figure 7a). The *R*_a,g_ of the polymers with increased BN sheet lateral size were almost identical (Figure 7b). This means that changing the lateral size of the BN sheet did not affect the distribution of the polymer chains. In addition, the *R_bulk_* of polymers with various sizes of BN sheets were almost the same (Figure 7c). The degree of entanglement of the polymer chains was also almost the same because there was no change of the distribution of the polymer chains. Figure 7d shows the phonon behaviors of the PEEK/BN composites. The phonon intensity in the low frequency range indicates the intensity of phonon vibration within a BN sheet [27]. The phonon vibration within BN sheets increased as the lateral length of BN sheets increased in the low frequency range from 30 THz to 60 THz. The increase of phonon vibration resulted in an improvement of the thermal conductivity of the PEEK/BN composite. In addition, the phonon intensity in the high frequency region (about 92 THz) at the interface between the polymer chains and fillers increased as the lateral length of the BN sheets increased [34]. The increase of phonon intensity at the interface between the polymer chains and BN sheets led to the improvement of the thermal conductivities of the PEEK/BN composites. 

**Figure 8:**
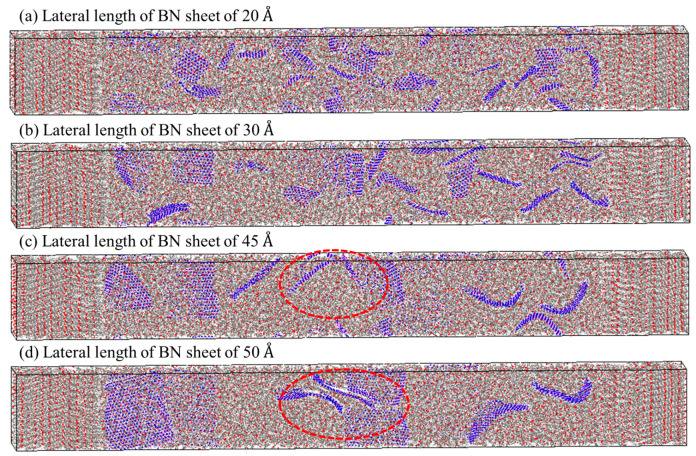
Equilibrium structure according to the lateral length of square BN sheets in PEEK/BN composites: (**a**) 20 Å (well-dispersed BN sheets), (**b**) 30 Å, (**c**) 45 Å, and (**d**) 50 Å (aggregated BN sheets as shown in the dotted red circle).

In addition, the dispersion of fillers affected the thermal conductivity of the composite [23]. The BN sheets in the composite models reinforced with BN sheets with lateral lengths of 20 Å and 30 Å were evenly dispersed, as shown in Figure 8a,b, respectively. The well-dispersed fillers facilitated more active heat transfer at the interface between the materials [23]. However, the thermal conductivities of the composites reinforced with BN sheets with lateral lengths of more than 30 Å gradually converged about 0.58 W/mK. The disconnection of the heat transfer path, due to aggregation of the BN sheets, leads to the convergence of the thermal conductivities (Figure 8c,d). These results mean that the improvement of thermal conductivity is limited by the aggregation of BN sheets, although the phonon transfer increased with the lateral size of the BN sheets. Therefore, the addition of BN sheets that are too large results in aggregation that obstructs the improvement of the thermal conductivity of the composite.

## 4. Conclusions

The important factors affecting the thermal conductivity of the semi-crystalline PEEK were revealed by the thermal transport simulations. The thermal conductivities of PEEK models with an amorphous chain with more than 70 repeat units were higher than the model with an amorphous chain of 10 repeat units. The long amorphous chains led to improved phonon transport by increasing the phonon vibration and collision probability. In addition, it was found that the wide distribution of long polymer chains with low entanglement induced long heat transfer paths. The thermal conductivity of the semi-crystalline polymer was related to the loop, tail, and bridge linkage conformations between the crystalline domain and the amorphous domain. The thermal conductivity of the model with high loop conformation content was enhanced by the increase of heat transfer paths, induced when both ends of the loop were connected with the crystalline domain. In addition, thermal conductivity increased as the bridge chain increased because both ends of the bridge chains were connected with the crystalline domains. However, an excessive content of bridge chains reduced thermal conductivity due to the entanglement of polymer chains. The thermal conductivities of the PEEK/BN composites were improved by better phonon transport after the addition of the BN sheets. In addition, the heat transfer path increased with the wide distribution of polymer chains along the surface of BN sheets. However, the thermal conductivity of the composite was related to the interfacial thermal transport between materials. Although phonon transfer improved as the lateral length of the BN sheets increased, the thermal conductivities of models containing BN sheets with lateral lengths of over 30 Å were converged due to the excessive aggregation of the BN sheets. These results mean that thermal conductivity was increased by the long heat transfer path induced by the long chain lengths, the high loop content, the optimal bridge content, and the addition of BN sheets. In addition, excessive bridge content and the aggregation of BN sheets caused polymer entanglement, resulting in low thermal transport. The simulation analyses of the structure of polymer are expected to assist the analysis of mechanisms for improving thermal properties of semi-crystalline polymer composites.

## Data Availability

The data presented in this study are available on request from the corresponding author.

## Supplementary Materials

The following supporting information can be downloaded at: https://www.mdpi.com/article/10.3390/polym15020450/s1, Figure S1: Energy of the bond stretching term using DFT and the N-DFF: (a) B–H bond and (b) N–H bond. Table S1: Parameters of the N-DFF used in the MD simulations. List of abbreviations. List of symbols.
