# Supplementary material for: Analysis of the Structure and the Thermal Conductivity of Semi-Crystalline Polyetheretherketone/Boron Nitride Sheet Composites Using All-Atom Molecular Dynamics Simulation
Source: Polymers (Basel). 2023 Jan 14;15(2):450. doi: 10.3390/polym15020450 (PMC9862992; doi:10.3390/polym15020450)
Supplement: Supplementary file 1 [file polymers-15-00450-s001.zip › polymers-2153285-supplementary.pdf]

**Supplementary Materials**

# Analysis of the Structure and the Thermal Conductivity of Semi-Crystalline Polyetheretherketone/Boron Nitride Sheet Composites Using All-Atom Molecular Dynamics Simulation

**Yuna Oh [1,2], Kwak Jin Bae [1], Yonjig Kim [2] and Jaesang Yu [1,*]**

**List of abbreviations**

| | |
|---|---|
| PEEK | Polyetheretherketone |
| BN | Boron nitride |
| MD | Molecular dynamics |
| h-BN | Hexagonal boron nitride |
| DSC | Differential scanning calorimeter |
| N-DFF | Newly proposed dreiding force field |
| DFT | Density functional theory |
| DFF | Dreiding force field |
| LLSF | Linear least square fitting |
| NPT | Isothermal-isobaric ensemble |
| RNEMD | Reverse non-equilibrium molecular dynamics |
| NVE | Microcanonical ensemble |
| PDOS | Phonon density of states |
| VACF | Velocity autocorrelation function |
| LAMMPS | Large-scale Atomic/Molecular Massively Parallel Simulator |
| RMSE | Root mean squared error |

## List of symbols

| | |
|---|---|
| $R_g$ | Radius of gyration (Å) |
| $R_{bulk}$ | Radius of gyration on the bulk polymer (Å) |
| $M$ | Total mass of the polymer chain (g/mol) |
| $m_i$ | Mass of the atom (g/mol) |
| $r_i$ | Coordinate of the atom |
| $r_{cm}$ | Coordinate of the center of mass on a polymer chain |
| $R_{a,g}$ | Average value of radius of gyration of the amorphous polymer chains (Å) |
| $R_{bulk}$ | Radius of gyration on the bulk polymer (Å) |
| $M_b$ | Total mass of the amorphous polymer chains (g/mol) |
| $r_{cm,bulk}$ | Coordinate of the center of mass on the bulk amorphous polymer |
| *ns* | Nanosecond |
| *fs* | Femtosecond |
| $K$ | Thermal conductivity (W/mK) |
| $J$ | Total heat flux (W/m$^2$) |
| $t$ | Heat transfer time (s) |
| $A$ | Cross-sectional area (m$^2$) |
| $m_h$ | Atomic masses of the heat source (g/mol) |
| $m_c$ | Atomic masses of the heat sink (g/mol) |
| $v$ | Velocity of atom (m/s) |
| $T$ | Temperature (K) |
| $\omega$ | Frequency (THz) |
| $\tau$ | Total integration time (s) |
| $v(t)$ | Velocity of atoms at time t (Å/ps) |
| $K_B$ | Force constants |
| $l$ | Bond length (Å) |

## Calculation of parameters for the bond stretching term

The force constants of the B-H and N-H bonds were obtained by linear least square fitting (LLSF) using the density functional theory (DFT) potential energy, which was calculated according to the bond length between boron atom and nitrogen atom in the unit cell. The bond potential energy of the newly proposed Dreiding force field (N-DFF) is expressed as

$$E_B = \sum_{Bonds} \frac{K_B}{2}(l - l_0)^2 \tag{S1}$$

where $K_B$ is the force constants on the bond stretching and $l$ is the bond length between two atoms. Figure S1 shows the bond potential energy obtained by the DFT and the molecular dynamics (MD) simulation using N-DFF. The root mean squared error (RMSE) values of the B-H and N-H bond terms calculated by the residuals between the DFT and MD potential energy were 0.004 and 0.015, respectively. These results mean that the force constants were accurately calculated with low error. Table S1 shows the force constants and the parameters of N-DFF. The sigma and epsilon of the B-H and N-H bonds on the Lennard-Jones function were calculated using the parameter values on the boron and nitrogen atoms of N-DFF, and the parameter values of the hydrogen atoms of the original Dreiding force field.

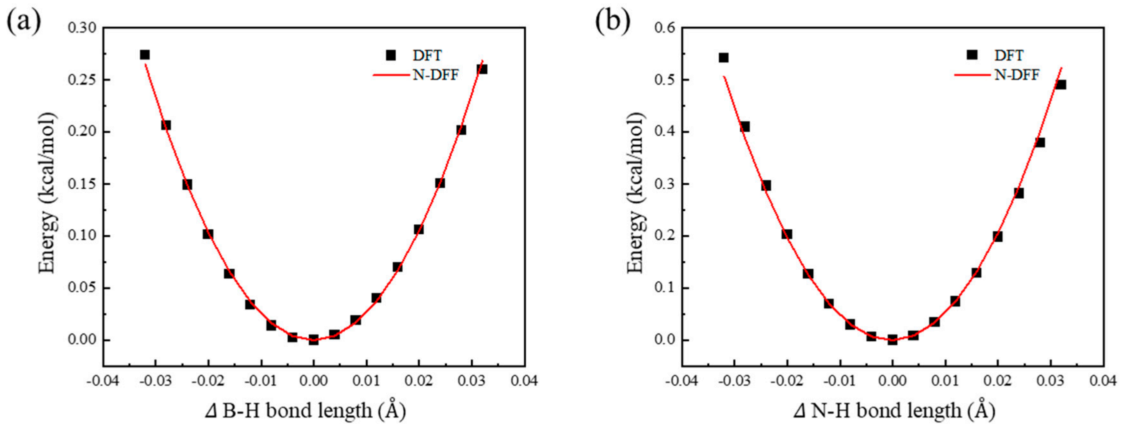


**Figure S1.** Energy of the bond stretching term using DFT and the N-DFF: (**a**) B-H bond and (**b**) N-H bond.

**Table S1.** Parameters of the N-DFF used in the MD simulations.

| **Bonding term** | **Value** | **Force constant (kcal/mol)** |
|---|---|---|
| B-H ($l_0$) | 1.202 Å | 521.102 |
| N-H ($l_0$) | 1.014 Å | 1006.20 |
| **Lennard-Jones** | **Sigma (Å)** | **Epsilon (kcal/mol)** |
| B-H | 3.2035 | 0.03309 |
| N-H | 2.9526 | 0.02480 |
